# Associations Between Pre-Fitting Factors and 2-Year Hearing Aid Use Persistence, Derived From Health Records and Post-Fitting Battery Order Data of 284,175 US Veterans

**DOI:** 10.1097/AUD.0000000000001694

**Published:** 2025-06-16

**Authors:** Graham Naylor, Lauren K. Dillard, Oliver Zobay, Gabrielle H. Saunders

**Affiliations:** 1 Hearing Sciences, Mental Health and Clinical Neurosciences, School of Medicine, University of Nottingham, Nottingham, UK; 2 NIHR Nottingham Biomedical Research Centre, Nottingham, UK; 3 Department of Otolaryngology–Head & Neck Surgery, Medical University of South Carolina, Charleston, South Carolina, USA; 4 VA Rehabilitation R&D, National Center for Rehabilitative Auditory Research, Portland, Oregon, USA; 5 Manchester Centre for Audiology and Deafness, School of Health Sciences, The University of Manchester, Manchester, UK.

**Keywords:** Electronic health record, Hearing aid, EHR, Persistence, Veteran

## Abstract

**Objectives:**

To examine associations between factors in domains of general health, hearing status, and demography, and subsequent long-term persistence of hearing aid (HA) use. By examining only non-modifiable factors available before HA fitting, we focus on potential indicators of a need for additional clinical effort to achieve satisfactory outcomes.

**Design:**

The initial dataset consisted of Electronic Health Records spanning 2012–2017, for all (731,231) patients with HA orders from U.S. Department of Veterans Affairs audiology between April 1, 2012 and October 31, 2014. Applying inclusion criteria (valid HA use persistence data, complete audiograms, age ≥50 years, audiometric pure-tone average (PTA) ≥25 dB HL, 5-year clearance period for health conditions) and excluding records with codes for cochlear implants, the final sample was 284,175 patients. Independent variables encompassed audiological (PTA, PTA asymmetry, audiogram slope, audiogram complexity, new versus experienced HA user), health (dementia, mild cognitive impairment, other mental health conditions, multimorbidity, in-patient episodes), and demographic (age, race, ethnicity, partnership status, income, urban–rural home location) domains. The outcome measure was HA use persistence at 2 years post-fitting, based on battery orders within 18 months preceding the 2-year mark. Multiple logistic regression modeling was applied with HA use persistence at 2 years post-fitting as outcome. Continuous variables were discretized; missing data were imputed.

**Results:**

After adjusting for covariates through the regression model, a significant positive association was found between PTA severity and HA use persistence, while PTA asymmetry, audiogram slope, and audiogram complexity were negatively associated with persistence. Being a new HA user, being diagnosed with dementia or other mental health conditions, and increased multimorbidity were all associated with reduced persistence. Persistence peaked at ages 70 to 79, and decreased for non-White races, Hispanic ethnicity, and those not married. No significant associations were found between persistence and tinnitus, urban–rural living, or mild cognitive impairment (when the model included dementia or other mental health conditions).

**Conclusions:**

Pre-fitting personal factors other than audiological ones have independent, and summatively major, influence on HA use persistence. While not being modifiable, some are potentially usable as flags for a differentiated approach to patient management.

## INTRODUCTION

Patients who discontinue use of their hearing aids (HAs) personify unachieved benefits and fruitless clinical and patient effort. The first step toward improving long-term HA use (here termed “persistence”) is to understand what drives and hinders it.

To address this aim, it pays to break it down into distinct components, whose premises and implications differ. To become a persistent HA user, a patient first has to take up a recommendation to obtain HAs and undergo HA fitting (“HA uptake”). The factors influencing HA uptake may differ from those influencing persistence given uptake. Hence, persistent HA use cannot be inferred from HA uptake/ownership, and there are two distinct and legitimate research questions deserving of attention. The first is, what factors influence uptake, and the second is, what factors influence persistence given uptake? Uptake is dependent only on factors present before HA fitting (i.e., preexisting personal factors and clinical process factors), whereas persistence may also be influenced by factors arising post-fitting (be they personal, further clinical processes, or based on experience with the HA).

In this study, we exclusively examine the effect of preexisting personal factors on persistence, given uptake. Such factors cannot be altered at the time of HA fitting, but they might indicate the need for extra clinical support to achieve positive outcomes, or even contraindications for HA fitting. Studies identifying factors that influence uptake are of limited direct consequence for the present study, in which the baseline point is after uptake. However, it is reasonable to expect that some factors influencing uptake will also influence persistence given uptake. The wide-ranging review by Knudsen et al. (2010) identified the following factors as likely to influence HA use: attitudes toward HA and toward own hearing loss, self-reported hearing problems, and manual dexterity. Variables identified in that review as unlikely to influence HA use were source of motivation, expectations, objective hearing loss, age, gender, and living arrangement. Mothemela et al. (2024) reviewed additional evidence arising from 2010 to 2023. Their review suggested that the following pre-fitting factors probably influence HA use: objective hearing loss, speech perception ability, self-reported hearing disability, bothersome tinnitus, race, attitude, personality, and education level. Equivocal results were observed for age, biological sex, and self-reported general health.

Most previous studies, including those reviewed by Knudsen et al. (2010) and Mothemela et al. (2024) have not isolated persistence outcomes from uptake outcomes. There are two exceptions. First, Humes (2023) examined data from 14,606 participants in the U.S. National Health Interview Study who had at some point obtained HAs. Subsequent “rejection” of HAs was found to be associated with lower age (below 30 versus over 50 years), absence of self-reported trouble hearing, annual income less than USD 50,000, male sex and low education level. Second, Gahlon et al. (2024) analyzed data from 666 participants in the U.S. National Health and Aging Trends Study. They found that “ceased” or “interrupted” HA use was associated with lower age at baseline (65 to 75 versus 75 to 85 years), income below the poverty line, education not beyond high school, and having a caregiver.

In this study, we interrogate a database of electronic health records (EHRs) collected routinely for clinical care over an 11-year period, to illuminate some pre-fitting factors associated with HA use persistence given uptake. It is not our intention to propose a specific model for use as a prediction tool with individual patients, but rather to identify potentially influential factors. Being a clinical dataset, the data for this study covers a finite set of pre-fitting factors: audiometry, in- and outpatient healthcare records (diagnoses and procedures) for all specialties, some demographics, and previous HA experience. Thus, our analysis is opportunistic with respect to the data source, and not exhaustive, but nevertheless rich with respect to plausible relations between pre-fitting patient variables and long-term HA use persistence.

Earlier studies from the same initial dataset have verified the validity of the dataset and demonstrated its potential for discovering novel associations, but did not include a multivariate model of persistence and therefore had limited ability to reveal complex relationships (Saunders et al. 2021). In addition, multivariate models including these variables were used to investigate (i) bidirectional associations between HA use persistence and dementia (Naylor et al. 2022) and (ii) the likelihood of long-term HA use persistence as a function of HA fitting laterality and better-ear vs. worse-ear audiometric severity and asymmetry (Zobay et al. 2023). The present study exploits the dataset in a different way; here we explore the extent to which diverse pre-fitting variables may influence HA use persistence even when other variables are accounted for.

For all the patients in our dataset, a pair of HAs (or a single aid in a minority of cases) had been ordered, and at least one subsequent order for HA batteries had been made, indicating that all patients had received their HA(s). Therefore, and important to reiterate, we are examining persistence (continued HA use) given HA uptake, not HA uptake itself.

## MATERIALS AND METHODS

This work was approved by the Institutional Review Board and the Research and Development Committee of the VA Portland Health Care System (Study No. 03566), Data Access Request Tracker (tracking number 2014-11-066-D-A04), and VA Patient Care Services.

### Analysis Approach

We present results from a multiple logistic regression model, which evaluates associations of predictor variables with the outcome of HA use persistence at 2 years post-fitting. This model was chosen because of its relative familiarity and straightforward interpretation. The model included main effects only, to avoid it becoming unhelpfully complex. All predictor variables that were continuous in their original form were discretized to facilitate the interpretation of nonlinear dependencies. For several discrete variables, some categories were combined for reasons of parsimony and to avoid small numbers in some subgroups of participants. Missing data for marital status, race, ethnicity, income, and urban/rural status (affecting 11.9% of patients) were filled in using hot-deck imputation (Kowarik & Templ 2016). All statistical analyses were performed with R software, version 4.4.1 (R Core Team 2024).

### Patient Sample

Our initial dataset consisted of EHRs for all (731,231) patients for whom HAs were ordered through VA audiology between April 1, 2012 and October 31, 2014. For all these patients, the dataset included demographic information, diagnostic (ICD-9/10) and procedural (CPT) codes related to health care provision in the VA system from January 1, 2007 to December 31, 2017, as well as records of the HA orders and of all HA batteries ordered through the VA health system for April 1, 2012 to December 31, 2017. A full description of the dataset and the pre-processing conducted can be found in Dillard et al. (2020) and Saunders et al. (2021).

For this study, the initial dataset was filtered according to the following inclusion criteria:

- Valid HA use persistence data (i.e., identifiable HA fitting session in EHR (Saunders et al. 2021) and not recorded as deceased by 2 years after fitting) (642,443/731,231).
- Air-conduction audiogram for left and right ears including all octave frequencies between 250 and 8000 Hz (555,993/731,231).
- Age at date of HA order ≥50 years. This focuses the study on patients with age-related hearing loss (707,894/731,231).
- Pure-tone average hearing threshold, mean across left and right ears (PTA; 500, 1000, 2000, 4000 Hz) ≥25 dB HL. This excludes patients with audiometrically normal hearing, who may have received HAs for reasons such as tinnitus relief (533,934/555,993 with valid PTA data).
- Clearance period: first recorded diagnosis of any health condition of interest (see later) at least 5 years before HA order. This ensures that patients have been in the VA Healthcare system for long enough that health condition diagnoses are likely to be captured (453,147/731,231).

Exclusion criteria were:

- Codes for cochlear implant anywhere in the EHR (1359/731,231).

Applying all the above filters simultaneously, the final sample was 284,175 patients. Mean, min-max, and standard deviations for age at HA order and PTA were 74.6, 50.0 to 105.0, 9.8 years and 50.8, 25 to 120 (upper limit is a pseudo-threshold for unmeasurable losses), 14.4 dB HL, respectively.

### Independent Variables

Given the panoply of variables available in the EHR, we applied the following criteria for their selection: (1) plausibility of an association with HA use persistence, that is, available argument of logic or prior evidence suggestive of a likely effect; and (2) potential for impact on service provision, that is, easily-assessed traits or distinct diagnoses which could flag a need for extra rehabilitative effort to achieve success or act as a contraindication for HA provision.

For each included variable described later, we only provide the rationale for its inclusion if its relevance is not self-evident.

#### Audiological Variables

**Fig. 1.**
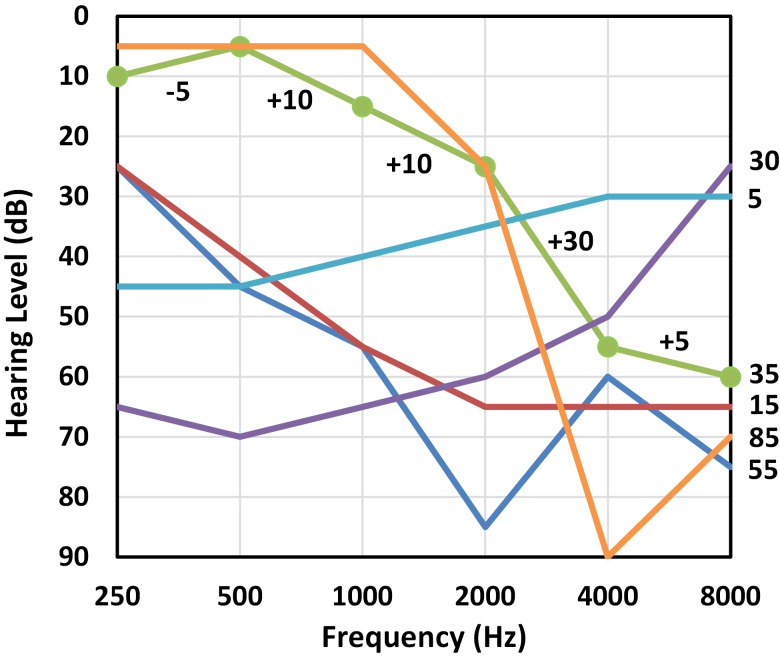
Example audiograms and their ΔOD values. To illustrate the calculation of ΔOD, the audiogram with ΔOD = 35 is annotated with the component octave-wise differences; the most positive is +30, and the least positive is −5, hence ΔOD = 35 dB HL/octave. ΔOD indicates audiogram complexity.

- PTA: PTA hearing threshold (500, 1000, 2000, 4000 Hz), averaged across left and right ears. Categorized as mild, moderate, severe, profound according to 25 ≥ PTA ≤ 40, 40 > PTA ≤ 60, 60 > PTA ≤ 80, PTA > 80 dB HL respectively.
- PTA asymmetry: Calculated as the absolute (unsigned) difference between the left and right PTA, and categorized into 0 to 9, 10 to 19, 20+ dB.
- Audiogram slope: Categorized as <0, 0 to 4, 5 to 9, 10 to 14, 15 to 19, 20+ dB/octave according to the slope of the regression line through all audiogram points (250 to 8000 Hz) when plotted on a logarithmic frequency axis, separately for each ear then averaged.
- Audiogram complexity (ΔOD): A crude indicator of the likely difficulty of achieving appropriate frequency shaping in an HA fitting, and calculated as the difference between the most and least positive octave-wise difference in threshold. For a standard-format audiogram in which all the points lie on a straight line, the octave-wise difference would be the same across all octaves, hence, ΔOD = 0. Testing showed that ΔOD is not strongly correlated with PTA or audiogram slope. Figure 1 shows example audiograms and their corresponding ΔOD values. ΔOD is calculated for each ear separately, then averaged, and was categorized into 0 to 9, 10 to 19, 20 to 29, 30 to 39, 40 to 49, 50 to 59, 60+ dB/octave.
- User type new versus experienced: The dataset included an explicit variable indicating whether or not the HA order in question was the patient’s first ever from the VA system.

#### Other Health Variables

These variables were included because they have all been observed individually to affect HA use persistence or compliance with healthcare interventions in general (see references cited per condition).

- Dementia (Cohen-Mansfield & Taylor 2004; Gregory et al. 2020; Naylor et al. 2022): Binary (yes/no), based on at least one diagnosis in the EHR before date of HA order (ICD-9 codes 290.XX [dementias], 294.1X [dementia in conditions classified elsewhere], 294.2X [dementia, unspecified], 331.0 [Alzheimer’s disease], 331.1X [frontotemporal dementia], 331.2 [senile degeneration of brain], 331.82 [dementia with Lewy bodies]).
- Mild cognitive impairment (MCI) (He et al. 2023): Binary (yes/no), based on at least one diagnosis in the EHR before date of HA order (ICD-9 code 331.83).
- Other mental health conditions (DiMatteo et al. 2000): Binary (yes/no), based on at least two diagnoses within 1 year before HA order amongst all ICD-9 codes listed in the HCUP classification scheme (Healthcare Cost and Utilization Project 2016a) as pertaining to body system 5 (mental disorders), other than the dementia codes specified above (NB: MCI is in body system 6—nervous system and sense organs). All diagnoses are accepted regardless of whether they are classified as chronic. The HCUP codes are mainly in the range 290 to 319 (ICD main category mental disorders) but also contain a few codes from other ICD main categories.
- Multimorbidity (Maffoni et al. 2020): The HCUP chronic condition indicator (Healthcare Cost and Utilization Project 2016b), based on at least two diagnoses per body system within 1 year before HA order, categorized as 0, 1, 2, 3, 4+ (number of body systems affected), after excluding codes used as above for dementia, MCI, and other mental health, and excluding hearing loss-related codes from body system 6.
- In-patient episode (Ma et al. 2020) within 5-year period before HA order: binary (yes/no).

#### Demographic Variables

- Age at date of HA order: While in many research contexts age acts as a catch-all proxy for unassessed age-related health conditions, here we account for many such conditions explicitly. It is worth examining whether age itself remains independently associated with the outcome measure (i.e., still a catch-all for predictor variables not included in our model). Age is categorized by decade: 50 to 59, 60 to 69, 70 to 79, 80 to 89, 90+ years.
- Race: There is evidence for race-related differences in uptake and use of hearing devices (Lee et al. 1991; Tomita et al. 2001; Bainbridge & Ramachandran 2014; Nieman et al. 2016). Here we can test whether there are also differences in persistence, given uptake. For the sake of parsimony and to avoid small cells, we condensed the EHR’s seven categories into three categories (White, Black or African American, Other), according to federal standards in force at the time of data collection (Federal Register 1997).
- Ethnicity: For the same reasons as above, we include ethnicity, recorded in the EHR as Hispanic versus non-Hispanic.
- Partnership status: Evidence suggests that the involvement of significant others in the hearing healthcare journey improves outcomes (Duijvestijn et al. 2003; Ellis et al. 2022), and Sawyer et al. (2019) found that those living alone were less likely to use an HA. For parsimony, we collapsed the EHR’s original five levels of this variable to four: married, separated or divorced, widowed, never married (i.e., we combined separated and divorced).
- Income: Hearing care, HA devices, repairs, and maintenance (including batteries) are all cost-free to eligible Veterans in the VA Healthcare System. Nevertheless, a patient’s income level may be associated with their HA use persistence (Gahlon et al. 2024) as it is to other interventions (Ji & Hong 2020). Here, we can examine whether any such effect is independent of other demographic variables, such as home location. On the basis of patient zipcodes, we approximate patient income using the zipcode median income data from the American Community Survey (ACS n.d.) for 2015–2019 (the closest time window to our data period), categorizing the result into one of four levels of annual income in USD (under 32k, 32–40k, 40–50k, over 50k).
- Urban–rural home location: Associations between home location and utilization of healthcare are known to be complex, as many influential demographic factors covary with the urban–rural axis (Cyr et al. 2019). It is unclear whether this axis is independently associated with hearing aid use persistence, given uptake. On the basis of the patients’ zipcodes recorded in the EHR, we calculate four levels (urban, large town, small town, rural) using the Rural–Urban-Commuting-Area codes (USDA n.d.; 2010 version used here).
- Sex and gender were excluded because the patient sample is 98.4% male.

### Outcome Measure—HA Use Persistence at 2 Years Post-Fitting

A measure of long-term HA use persistence was computed based on the number of battery orders made by the patient, as detailed in Zobay et al. (2021) and Saunders et al. (2021). Briefly, an HA recipient is considered to be a persistent HA user at 2 years after their HA fitting if they had at least one battery order within the 18-month period preceding the 2-year mark. This outcome measure is robust against moderate variations of either the endpoint time or battery-ordering time window length (Zobay et al. 2021).

## RESULTS

Table 1 shows patient characteristics and the results from the full model.

**TABLE 1.** Patient characteristics and factors associated with hearing aid use persistence from unadjusted and adjusted models

Predictor	Level	N	Proportion (%)	Persistence (%)	OR Unadj	CI (95%) Unadj	OR Adj	CI (95%) Adj	*p* Value Adj
Intercept		284,175	100	65.3	1.88	1.87–1.90	2.57	2.45–2.69	<0.001
Age (yrs)	50–59	16,601	5.8	50.8	1		1		
	60–69	90,675	31.9	58.4	1.36	1.32–1.41	1.1	1.06–1.14	<0.001
	70–79	80,861	28.5	67.9	2.06	1.99–2.13	1.25	1.21–1.30	<0.001
	80–89	82,000	28.9	72.1	2.5	2.42–2.59	1.18	1.13–1.22	<0.001
	90+	14,038	4.9	73.1	2.63	2.51–2.76	1.04	0.99–1.10	0.128
PTA	Mild	72,238	25.4	51.0	1		1		
	Moderate	143,755	50.6	66.3	1.89	1.85–1.92	1.51	1.48–1.54	<0.001
	Severe	58,452	20.6	78.2	3.45	3.37–3.54	2.45	2.38–2.52	<0.001
	Profound	9730	3.4	79.7	3.77	3.58–3.97	2.73	2.59–2.89	<0.001
PTA asymmetry	0–9	211,412	74.4	65.7	1		1		
	10–19	46,512	16.4	65.2	0.98	0.96–1.00	0.95	0.93–0.97	<0.001
	20+	26,251	9.2	62.4	0.86	0.84–0.89	0.69	0.67–0.71	<0.001
Audiogram slope	<0	1918	0.7	63.5	0.94	0.85–1.03	1.2	1.08–1.33	<0.001
	0–4	20,624	7.3	66.4	1.06	1.03–1.10	1.11	1.07–1.15	<0.001
	5–9	83,164	29.3	66.7	1.08	1.06–1.10	1.07	1.04–1.09	<0.001
	10–14	113,946	40.1	65.0	1		1		
	15–19	54,244	19.1	64.1	0.96	0.94–0.98	0.91	0.89–0.93	<0.001
	20+	10,279	3.6	62.8	0.91	0.87–0.95	0.76	0.72–0.79	<0.001
ΔOD	0–9	1477	0.5	70.8	1.08	0.97–1.21	0.98	0.87–1.11	0.779
	10–19	33,368	11.7	70.8	1.08	1.05–1.11	1.04	1.01–1.07	<0.001
	20–29	79,144	27.9	69.1	1		1		
	30–39	80,838	28.5	66.1	0.87	0.86–0.89	0.93	0.91–0.95	<0.001
	40–49	51,192	18.0	61.5	0.72	0.70–0.73	0.83	0.81–0.85	<0.001
	50–59	24,144	8.5	57.8	0.61	0.59–0.63	0.76	0.74–0.78	<0.001
	60+	14,012	4.9	52.6	0.5	0.48–0.52	0.65	0.63–0.68	<0.001
Tinnitus	No	188,022	66.2	66.3	1		1		
	Yes	96,153	33.8	63.4	0.88	0.86–0.89	0.99	0.97–1.01	0.266
User type	Experienced	174,220	61.3	72.6	1		1		
	New	109,955	38.7	53.8	0.44	0.43–0.45	0.56	0.55–0.56	<0.001
Dementia	No	276,473	97.3	65.6	1		1		
	Yes	7702	2.7	55.7	0.66	0.63–0.69	0.6	0.57–0.63	<0.001
MCI	No	279,253	98.3	65.4	1		1		
	Yes	4922	1.7	60.6	0.81	0.77–0.86	0.94	0.89–1.01	0.069
Other mental	No	198,109	69.7	69.2	1		1		
	Yes	86,066	30.3	56.5	0.58	0.57–0.59	0.77	0.75–0.78	<0.001
Multi–morbidity	0	39,528	13.9	72.0	1		1		
	1	49,799	17.5	67.9	0.82	0.80–0.85	0.94	0.91–0.96	<0.001
	2	61,639	21.7	65.9	0.75	0.73–0.77	0.9	0.88–0.93	<0.001
	3	55,302	19.5	63.5	0.68	0.66–0.70	0.86	0.84–0.89	<0.001
	4+	77,907	27.4	61.2	0.62	0.60–0.63	0.86	0.84–0.89	<0.001
In-patient visits	No	226,179	79.6	67.5	1		1		
	Yes	57,996	20.4	56.7	0.63	0.62–0.64	0.81	0.79–0.83	<0.001
Race	White	261,185	91.9	66.6	1		1		
	Other	6693	2.4	59.2	0.73	0.69–0.77	0.76	0.72–0.80	<0.001
	Black	16,297	5.7	47.0	0.45	0.43–0.46	0.56	0.54–0.58	<0.001
Ethnicity	Non-Hispanic	275,606	97.0	65.7	1		1		
	Hispanic	8569	3.0	52.7	0.58	0.56–0.61	0.64	0.61–0.67	<0.001
Partnership	Married	204,467	72.0	68.3	1		1		
	Widowed	25,686	9.0	65.8	0.89	0.87–0.92	0.76	0.73–0.78	<0.001
	Div./Sep.	44,906	15.8	54.3	0.55	0.54–0.56	0.68	0.67–0.70	<0.001
	Never married	9116	3.2	51.7	0.5	0.48–0.52	0.62	0.59–0.65	<0.001
Income USD (per yr)	>50k	194,146	68.3	66.9	1		1		
	40–50k	59,588	21.0	63.6	0.87	0.85–0.88	0.9	0.88–0.92	<0.001
	32–40k	23,332	8.2	60.0	0.74	0.72–0.76	0.81	0.78–0.83	<0.001
	<32k	7109	2.5	54.3	0.59	0.56–0.62	0.76	0.72–0.80	<0.001
Urban–rural	Urban	213,450	75.1	65.4	1		1		
	Large town	34,623	12.2	65.0	0.98	0.96–1.01	0.99	0.96–1.01	0.306
	Small town	19,664	6.9	64.9	0.98	0.95–1.01	0.99	0.96–1.03	0.701
	Rural	16,438	5.8	66.2	1.04	1.01–1.08	1.04	1.00–1.07	0.052
AUC	0.6858								
Pseudo-*R*^2^	0.1259								

Sex and gender are not included in the model as 98.4% of patients are male. OR =1 is the referent value.

Adj, adjusted; AUC, area under the curve; CI, confidence interval; MCI, mild cognitive impairment; OR, odds ratio; Unadj, unadjusted; ΔOD, audiogram complexity metric.

**Fig. 2.**
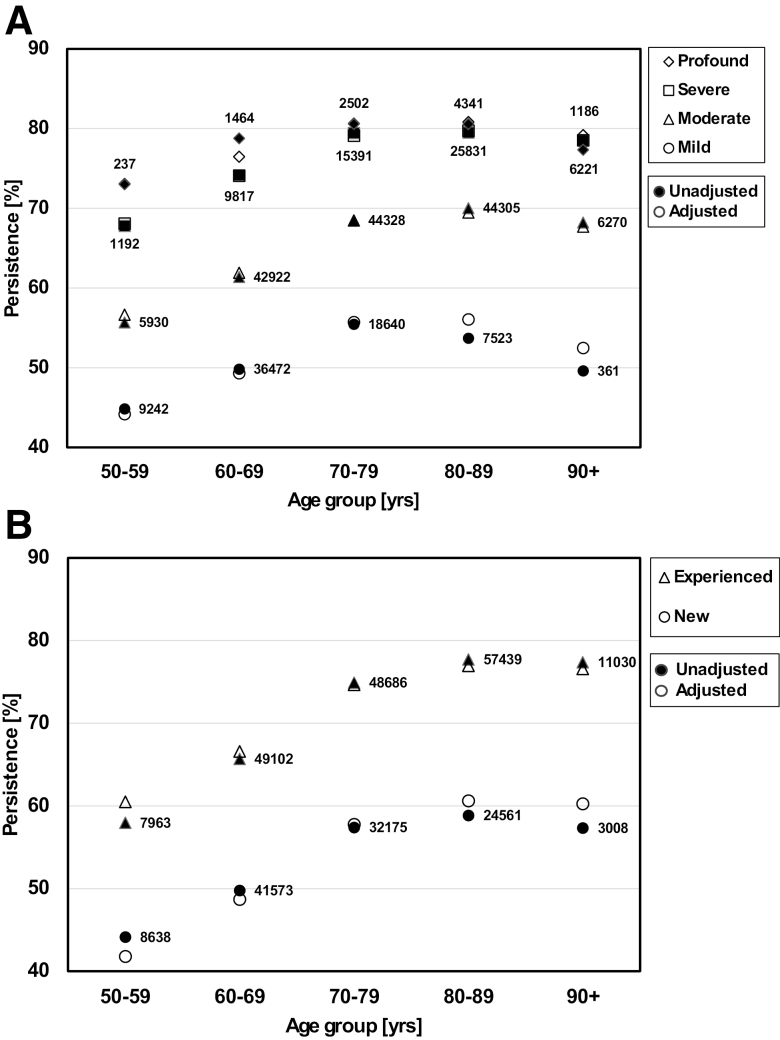
Unadjusted and adjusted HA use persistence rate against age decade, with (A) hearing loss severity and (B) user-type variables as parameters (symbol shapes). Unadjusted (filled symbols): mean of the observed persistence status across all patients in a cell defined by the levels of the crossed variables. Adjusted (open symbols): mean of the probabilities of persistence predicted by the fitted regression model. Numbers: number of observations in a given cell. HA, hearing loss.

**Fig. 3.**
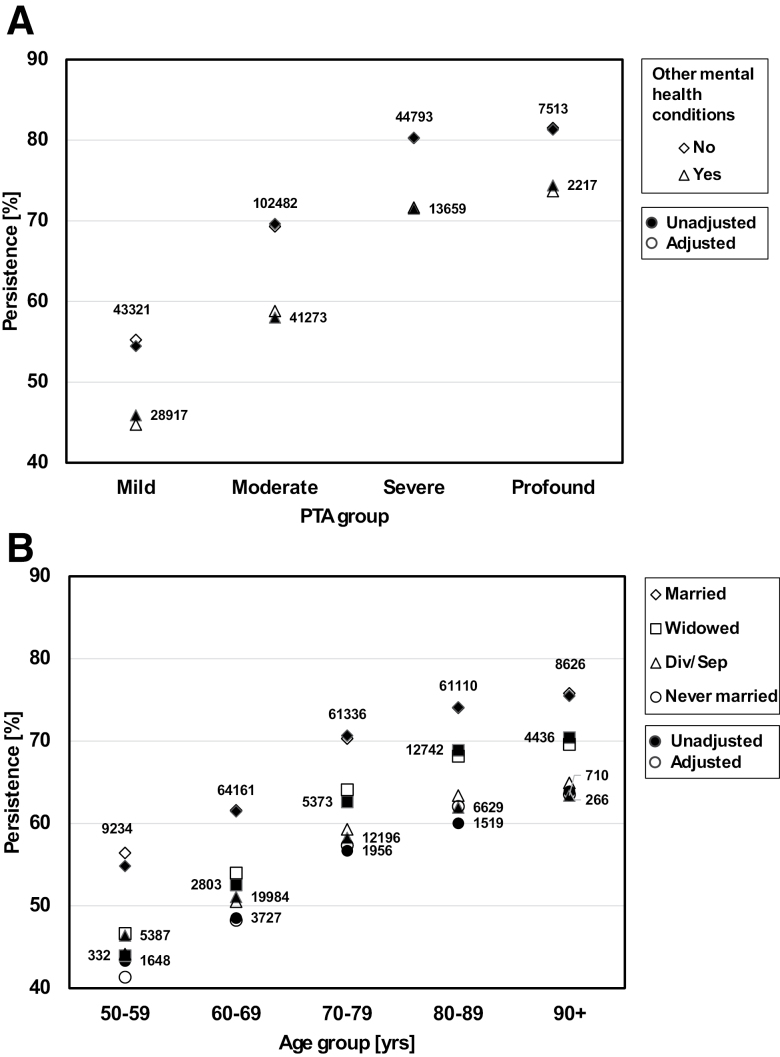
Unadjusted and adjusted HA use persistence rates. A, Against hearing loss severity and presence of a diagnosis of “other mental health condition.”. B, Against age decade and partnership status. Unadjusted (filled symbols): mean of the observed persistence status across all patients in a cell defined by the levels of the crossed variables. Adjusted (open symbols): mean of the probabilities of persistence predicted by the fitted regression model. Numbers: number of observations in a given cell. HA, hearing loss.

Figure 2 shows the variation of observed (unadjusted) and modeled (adjusted) HA use persistence against age decade, with (A) hearing loss severity and (B) user-type variables as parameters. In Figure 3, unadjusted and adjusted persistence are shown against (A) age decade and partnership status, and (B) hearing loss severity and presence of a diagnosis of “other mental health condition” as defined earlier.

For all variables except age and audiogram slope, raw and adjusted ORs (Table 1) show the same trends across levels of the variable. The graphical examples in Figures 2 and 3 show model-adjusted persistence rates that, in most cases, provide good approximations to the observed rates. This indicates that our main effects-only model has not missed substantial interactive components.

The goodness-of-fit statistics for the full model (Table 1) are area under the curve = 0.6861 and Pseudo-*R*^2^ = 0.1264. These demonstrate that the model performs at well above chance level, but emphasize that while the model may identify risk factors, there remains a lot of unexplained variance.

## DISCUSSION

### General Discussion

In this study, we used EHRs to model HA use persistence at 2 years post-fitting among 284,175 US Veterans who all had obtained HAs through the VA healthcare system. We determined that preexisting personal factors from demographic, audiological, and other health domains were independently associated with HA use persistence.

The effects of most individual factors are in line with expectations. Modeled persistence (adjusted OR) increases strongly with PTA severity, and decreases markedly when PTA asymmetry exceeds about 20 dB HL. Persistence decreases continuously with audiogram slope, with highest persistence for negative slope and lowest for slopes exceeding 20 dB/octave. Audiogram complexity (ΔOD) values below about 40 dB/octave have little effect, but more highly complex audiograms substantially reduce persistence. The presence vs. absence of a tinnitus diagnosis has no association with persistence; however, the prevalence distributions of tinnitus in samples of Veterans diverge from those usually found in population samples (Folmer et al. 2011). New HA recipients are much less likely to remain persistent users than patients who are already experienced HA users.

Persistence reduces strongly with prevalent dementia, but MCI shows no effect. In sensitivity analyses, removal of either dementia or other mental health from the model caused the adjusted OR for MCI to fall significantly below unity. This may mean that co-occurrence patterns of MCI, Dementia, and other mental health diagnoses in our sample result in interactions that our main effects-only model cannot capture, or that some effects of multicollinearity are at play. The presence of mental health conditions (other than dementia and MCI) has a considerable effect on persistence, reducing it by about ten percentage points regardless of PTA (Fig. 3A). Relative to patients with no multimorbidities, persistence decreases gradually as the number of body systems with diagnoses increases. While the multimorbidity variable is insensitive to the severity of diagnosed conditions, the in-patient episodes variable may be considered a proxy for the presence of a serious condition, and indeed, it too shows a sizeable effect on persistence.

Persistence increases with age above 50 years, peaking at 70 to 79 years before falling again, such that patients 90+ years old show persistence similar to that of patients who are 50 to 59 years old. Therefore, it appears that age-related effects are still present, despite controlling for a diverse array of variables concerning conditions associated with age. We cannot be certain why persistence peaks at 70 to 79 years. It seems plausible that decline toward older age may be driven by aspects of increased frailty and changes in social engagement not captured in our data. Because all the patients in our data have taken up HAs, the decline toward younger age cannot be explained as an effect of perceived social acceptability of HA uptake, or delay in uptake (Simpson et al. 2019). However, it might be explained in terms of stigma relating to actual use of HAs. In any case, it appears that the benefit/burden ratio is maximized for the 70 to 79-year age group. The non-monotonic association with age found here is consistent with that found by Gahlon et al. (2024), and may explain why previous reviews (Knudsen et al. 2010; Mothemela et al. 2024) show inconsistent trends across studies covering differing age ranges.

Race and ethnicity show strong associations with persistence, in line with previous evidence showing patients of non-White race or Hispanic ethnicity to have substantially less favorable outcomes for both HA uptake and HA use (Lee et al. 1991; Tomita et al. 2001; Bainbridge & Ramachandran 2014; Nieman et al. 2016). The results for the partnership variable (Fig. 3B) indicate that patients who are presumably living with others (i.e., married) are more persistent than patients in other partnership categories who may not be living with others. This is consistent with the result found by Sawyer et al. (2019). The effect of income on persistence is significant, and consistent with results found by Humes (2023) and Gahlon et al. (2024). Our results suggest a gradual reduction of persistence as income falls, rather than a threshold-like effect. The urban–rural factor shows no effect on persistence after controlling for other available demographic factors. This result may not generalize to non-Veteran populations, as the geographical distribution of Veterans’ home locations, and reasons for residing there, may not be representative of the general population (Holder 2017). Furthermore, there might be an effect of rurality on uptake (driven by accessibility of clinical facilities), but not on persistence (as batteries can be delivered to the home).

Overall, we show that even when combining a considerable number of variables, they retain independent effects on the outcome of HA use persistence. To summarize in a single sentence, one might say that anything which is generally known to be detrimental to health or life chances is also associated with reduced long-term HA use persistence. This aligns with the concept of Patient Complexity (Shippee et al. 2012), whereby complicating patient-related factors impact care and outcomes via the (im)balance between patient “workload” and “capacity.” Both illness and treatment contribute to “workload” (and potentially “capacity”) via feedback loops, with the implication that unless treatment improves the balance between “workload” and “capacity” (total across all aspects of the patient), the balance will gradually worsen, and treatment is at risk of being abandoned by the patient. In the present case, the burden of superficially irrelevant health conditions and/or their treatment can act as “a thumb on the scales,” such that HA use needs to provide an exceptional benefit-to-burden ratio if use is to persist.

### Assessing Relative Importance of Variables and Implications for Clinical Processes

Besides PTA, a few variables stand out as having relatively strong influence on modeled HA use persistence:

- PTA asymmetry >20 dB and audiogram complexity (ΔOD) >60 dB/octave: These factors are familiar drivers of decision-making and fine-tuning processes.
- New HA users: It is logical that the best basis for future persistence is past persistence (i.e., experience), but the strong influence this factor possesses is a reminder of the potential value of differentiated clinical efforts for new vs. experienced users. This highly influential factor is also extremely easy to ascertain during the HA assessment and fitting process.
- Patient race (Black) and ethnicity (Hispanic): These are also easy to ascertain, but fostering long-term persistence for these groups is likely to require complex interventions involving community engagement, diversity training, and increasing trust (Marrone et al. 2017; Shen et al. 2018; Robertson et al. 2021).
- Being divorced/separated, never married, or widowed. This “not living with others” factor is easy to ascertain clinically, and the value of partner support (whether through extrinsic or intrinsic motivation) for HA adoption and satisfaction has been shown previously (Hickson et al. 2014; Singh et al. 2015). It is not clear how best to motivate the patient who lives alone to use HAs, but recognizing the likely patient need is a first step.

### Strengths and Limitations

Strengths of this study include the large sample size, the inclusion of variables from diverse domains, the availability of a long-term outcome measure, and that the data arises from routine clinical practice across a large healthcare system with its inevitable local variations in population and practice. While routine clinical data inevitably includes more coding errors and inconsistencies than research data, this will largely be random variance, such that any effects found will be underestimated rather than driven by bias.

Amongst the study’s limitations, it is important to note that the list of predictor variables was determined by what was available, not what one ab initio might wish to examine. While the included variables are diverse, some potentially influential domains (e.g., attitudinal variables relatable to stigma) were unavailable.

The patient sample’s provenance in the VA healthcare system leads to some inevitable imbalances. Primary among these is that 98.4% of patients were male. While gender differences might alter the magnitude of effect of some demographic factors, it seems unlikely that the overall picture would be much different. The VA context also means that the patient sample is not fully representative of the general clinical population with respect to patterns of health and demographics. Nevertheless, we may expect the individual associations between factors and outcome to be indicative of likely presence in a general clinical population sample. As with many previous studies of HA use, caution is also warranted regarding the generalisability of the current findings to general population (as opposed to clinical) samples.

All the effects found here appear plausible, and there is little reason to think that any of them are chance results of the particular dataset. However, the associations found cannot be taken as generalizable to all other contexts, without being tested in datasets of different provenance.

The dataset used in this study covers the years 2012–2017 for HA use persistence, and therefore the likely currency of our results deserves comment. As HA technology is not an explicit variable under study, there are no obvious ways in which it might change the relations observed here, other than their numerical magnitudes. If anything, we might expect a more current dataset to show less dependence of HA use persistence on audiometric factors, as newer technology is presumably better at dealing with those (e.g., frequency shaping, ear-to-ear coordination of processing).

Because the patient sample only included people who had at least one HA order, all patients had passed through the uptake gate in their treatment pathway. Therefore, we cannot conclude anything about how the variables studied might affect HA uptake, only HA use given HA uptake. On the other hand, we can be sure that effects on HA uptake and on HA use persistence are not being conflated.

## CONCLUSION

Personal pre-fitting factors other than audiological ones have independent, and summatively major, influence on HA use persistence. While not being modifiable, some are potentially usable as flags for a differentiated approach to patient management. Our data suggests that particularly influential variables include previous HA use, large asymmetry of hearing loss, complex audiogram shape, race/ethnicity, and partnership status. All of these are easily ascertained at the time of HA fitting, but the management differentiations they might motivate are likely to vary widely in character.

Further studies are needed to test the replication of these effects in other patient samples, and to examine the additional effects of post-fitting variables such as follow-up sessions.

## ACKNOWLEDGMENTS

The authors thank Kevin Quitmeyer, ShienPei Silverman, Kelly Reavis, Erin Robling, Dawn Konrad-Martin, and M. Patrick Feeney for their support throughout the study, and Tim Beechey for alerting us to the relevance of the Patient Complexity concept.
